# Lived Experiences of Adoptive Parents Raising Children with Fetal Alcohol Spectrum Disorders in Israel: A Qualitative Study

**DOI:** 10.3390/children13050597

**Published:** 2026-04-26

**Authors:** Natalia Zwilling, Liat Hen-Herbst, Liat Korn

**Affiliations:** 1 Department of Health Systems Management, Faculty of Health Sciences, Ariel University, 3 Kiryat Hamada St., Ariel 40700, Israel; natalia.zwilling@msmail.ariel.ac.il (N.Z.); liatk@ariel.ac.il (L.K.); 2 Department of Occupational Therapy, Faculty of Health Sciences, Ariel University, 3 Kiryat Hamada St., Ariel 40700, Israel

**Keywords:** adoptive parents, caregiver burden, fetal alcohol spectrum disorder, FASD, Israel, public health policy, qualitative research

## Abstract

**Highlights:**

**What are the main findings?**

- Fluctuating child functioning creates ongoing uncertainty for families.
- Parents use coping strategies, but formal support remains insufficient.

**What is the implication of the main finding?**

- Findings highlight the need for coordinated diagnostic and educational services.
- Ecological and family systems perspectives advancing understanding of FASD in adoptive contexts.

**Abstract:**

**Background**: Fetal alcohol spectrum disorders (FASDs) are lifelong neurodevelopmental conditions resulting from prenatal alcohol exposure. Despite high FASD rates in high-risk adoptive populations, little is known about the experiences of adoptive parents in Israel, where underdiagnosis and limited professional awareness persist. Drawing on ecological and family systems theory, this study explored the daily challenges, coping strategies, and service-related barriers encountered by adoptive parents raising children with FASD. **Methods**: Using a qualitative, exploratory design, semi-structured interviews were conducted with 12 adoptive parents of children aged 9–18 years with a parent-reported clinical diagnosis of FASD made by a healthcare professional. The interviews focused on child-related difficulties, coping mechanisms, diagnostic experiences, and interactions with health, education, and welfare systems. The data were analysed using reflexive thematic analysis. **Results**: Five themes were identified: (a) child-related neurodevelopmental and behavioral challenges, (b) emotional and practical caregiving burden, (c) systemic barriers, including limited professional knowledge and fragmented diagnostic pathways, (d) coping strategies and family strengths, and (e) unmet needs and recommendations. Parents described fluctuating child functioning, chronic caregiving demands, and significant gaps in diagnostic and educational support. **Conclusions**: Adoptive parents raising children with FASD face substantial daily challenges compounded by systemic limitations. This study’s findings underscore the need for improved professional training, clearer diagnostic pathways, FASD-informed educational practices, and coordinated multidisciplinary services. These results provide direction for policy and service development to improve support for families affected by FASD.

## 1. Introduction

Fetal alcohol spectrum disorders (FASDs) represent a range of lifelong neurodevelopmental conditions caused by prenatal alcohol exposure. First recognized by Lemoine et al. [1] and later described by Jones et al. [2], FASDs encompass a range of physical, cognitive, behavioral, and adaptive challenges that vary in type and severity [3]. Common neurodevelopmental difficulties include impairments in executive functioning, learning, memory, attention regulation, sensory processing, and adaptive behaviors, such as social skills and daily living abilities [4,5]. Globally, FASDs are considered the most common potentially preventable condition correlated with developmental disabilities and birth anomalies [6], with prevalence estimates exceeding 1% of children in the general population [7]. Nevertheless, reporting varies considerably across countries, shaped by differences in diagnostic infrastructure, professional awareness, and surveillance systems [8].

In Israel, official documentation of FASD remains significantly lower than in other Western countries. Between 2005 and 2019, only 184 cases were reported by three of the four national health maintenance organizations [9]. This marked underdiagnosis contrasts with maternal alcohol consumption rates that parallel global patterns: approximately 70% of mothers consumed alcohol in the 3 months before conception, and 10% to 12% after conception [10,11].

Evidence from Israeli high-risk groups strongly suggests that the actual prevalence of FASD in Israel is much higher than officially reported. Studies have identified FASD in 15% of foster and early-adopted infants aged 0 to 2 years [12] and in 63% of pre-adopted and fostered children with documented prenatal alcohol exposure, who exhibited FASD-related characteristics at a mean age of 5.7 years [13]. These populations often include children who have experienced prenatal substance exposure, neglect, or family instability, factors that may lead to placement in foster care or adoption and are also associated with an increased risk of FASD. These findings are consistent with international research demonstrating elevated FASD rates among children in out-of-home care, adopted children, and those with prenatal risk factors. For example, meta-analytic estimates indicated that approximately 16.9% of children in care meet diagnostic criteria for FASD [14], and studies in Western Europe report prevalence rates of 30% to 70% among children adopted from Russia and Ukraine [15]. Together, these data suggest that Israel mirrors global patterns of underdiagnosis, particularly within populations characterized by early adversity and multiple risk exposures.

Children with FASD frequently experience secondary difficulties, such as mental health problems [16], learning challenges, behavioral problems [17], and struggles in daily functioning [18]. These difficulties tend to emerge when neurodevelopmental vulnerabilities meet environments that fail to recognize or accommodate the specific needs associated with FASD. Over time, misunderstandings of the disorder, inconsistent expectations, delayed diagnosis, and insufficient support in educational and health care systems can amplify the risk of developing secondary mental health and behavioral problems [19].

The impact of FASD extends beyond the child; it profoundly affects parents, siblings, and the family system as a whole [20]. Parents often describe parenting as an “unpredictable and exhausting journey” that requires constant vigilance, creativity, and adaptation to their child’s changing needs [21,22]. Across multiple studies, caregivers reported chronic stress, emotional exhaustion, frustration, and feelings of guilt or inadequacy, often stemming from societal stigma and a lack of understanding from professionals and the public [23,24,25].

Furthermore, parents experience isolation from extended family or peers, citing judgment, misunderstanding, or disbelief regarding the legitimacy of their child’s diagnosis [26,27]. The constant need to advocate for services and recognition has been described as both burdensome and empowering, fostering a sense of expertise and purpose over time [28,29]. However, despite the heavy emotional and practical demands, many caregivers also demonstrate remarkable resilience. Studies have highlighted parents’ ability to find meaning, hope, and emotional growth through advocacy, connection with support groups, and reframing of expectations [30,31,32]. This coexistence of strain and strength underscores the complexity of parenting a child with FASD and highlights the urgent need for responsive, family-centered professional support systems.

Importantly, while a substantial body of research has been conducted in high-income countries, emerging studies from Global South contexts highlight additional caregiving challenges under conditions of structural adversity, limited access to services, and resource constraints [33]. These perspectives underscore the importance of considering broader socio-economic and cultural contexts when examining caregiving experiences.

The present study draws on ecological systems theory [34] and family systems theory [35], which emphasize that interactions across individual, relational, community, and societal contexts shape parenting a child with a developmental disability. From an ecological perspective, caregiving experiences are understood as shaped by interactions across multiple levels, including the child’s characteristics, family dynamics, educational settings, healthcare systems, and broader policy environments [34]. Family systems theory further highlights how the child’s needs and behaviors influence family roles, routines, and relational patterns, requiring ongoing adaptation within the family unit [35]. Together, these frameworks informed both the study design and the analytic approach, guiding attention to the interplay between individual, familial, and systemic factors in shaping parents’ lived experiences. This perspective allows for an examination of how Israeli adoptive parents’ experiences are influenced not only by their child’s needs and characteristics but also by broader cultural beliefs, service structures, and policy environments.

### Study Objective

Despite the extensive international literature on FASD and caregiving, no qualitative studies have examined the lived experiences of adoptive parents raising children with FASD in Israel. Given the high prevalence of FASD in high-risk groups, marked by underdiagnosis and the unique complexities of adoptive parenting, this represents a significant gap.

Therefore, this study aims to (a) describe the challenges adoptive parents encounter in their daily lives with children diagnosed with FASD, (b) identify the coping strategies and sources of support these parents use, and (c) examine how social, cultural, and systemic factors in Israel influence parental experiences and access to services. In addition, the study situates these experiences in relation to both globally documented patterns of FASD caregiving and the specific features of the Israeli service and policy environment. By giving voice to an underrepresented group of caregivers, this study addresses a significant gap in the Israeli and international literature. The findings can inform professional training, guide the development of family-centered interventions, and strengthen multidisciplinary diagnostic frameworks. They may also assist policymakers in improving public awareness, resource allocation, and long-term support for adoptive families affected by FASD.

Accordingly, the overarching research question was as follows: how do adoptive parents in Israel experience raising a child with FASD within family and service-system contexts?

## 2. Materials and Methods

### 2.1. Study Design

This study used a qualitative, exploratory design with semi-structured, in-depth interviews to explore the experiences of adoptive parents raising children diagnosed with FASD in Israel. A thematic analysis approach [36] guided the analytic process, allowing the identification of patterns and themes across participants’ narratives.

The Ariel University Institutional Review Board (No. AU-HEA-LK-20240515 on 15 May 2024) provided ethical approval for the study, which adhered to the Consolidated Criteria for Reporting Qualitative Research guidelines [37] to enhance methodological transparency. Participation in the study was entirely voluntary, and all data were handled in strict confidence. All participants provided written informed consent before participation. They were informed of their right to decline to answer questions or withdraw at any time without consequences. To ensure confidentiality, pseudonyms were assigned, and identifying information was removed during transcription and analysis.

Given the sensitive nature of the research, which included discussions of behavioral challenges, violence, and parental distress, participants were explicitly informed of their right to skip questions or pause the interview at any time. The interviewer was attentive to signs of emotional discomfort and offered breaks when needed. Participants were also provided with contact information for relevant professional services and parent advocacy organizations, should they require assistance beyond the research context.

### 2.2. Participants and Recruitment

Twelve adoptive parents participated in the study, including ten mothers and two fathers. Both parents were present at one of the 11 interviews. The sample size of 12 was considered sufficient to achieve thematic saturation, as the interview data became redundant and no new themes emerged regarding the central research questions after the 10th interview. Parents ranged in age from 50 to 62 years, and their children were aged 9 to 18. All children had been adopted by the age of 3 years. Most (n = 9) were born in Eastern European countries, and two were born and adopted in Israel. The average duration of parenthood was 12.97 years (SD = 2.78). In families with more than one child diagnosed with FASD (P9/10, P11, and P12), the sociodemographic information presented in Table 1 pertains to a single focal child per family. This child was selected based on parental choice or because the siblings were over 18. The interviews, however, could include references to experiences with more than one child, where relevant. Half of the interviewees were married, four were divorced, and two of them were single parents.

The participants were recruited in two phases. First, announcements were shared through Israeli parent support groups for adoptive families. Additional participants were then recruited through snowball sampling, with parents referring to others in their social networks. Inclusion criteria were (a) adoptive parents of at least one child aged 9 to 18 years with a parent-reported clinical diagnosis of FASD, made by a healthcare professional, at least 2 years before the study, (b) proficiency in Hebrew, (c) willingness to participate in an in-depth interview, and (d) provision of written informed consent. Exclusion criteria were nonadoptive parents of children diagnosed with FASD or parents of children with severe neurological disorders not attributable to FASD (e.g., epilepsy, Down syndrome, severe cerebral palsy).

The sample size of 12 participants was determined to be sufficient to achieve thematic saturation, as no substantially new themes emerged after approximately the 10th interview, and subsequent interviews confirmed and deepened existing themes. Recruitment was concluded when thematic saturation was reached. Given the specificity of the sample and the depth of the data collected, the sample was considered sufficient to address the study aims, consistent with the concept of information power [38].

### 2.3. Data Collection

The interviews were conducted in person between May and August 2024 and lasted 45–120 min. A semi-structured interview guide was used to facilitate open-ended discussion. Topics included the child’s strengths and challenges, family coping strategies, adoption-related experiences, the diagnostic process, interactions with health and educational systems, and sources of formal and informal support. Participants were encouraged to share concrete examples from their everyday lives and describe positive and challenging experiences in as much detail as they wished.

No prior relationship existed between the interviewer and participants before recruitment. The interview guide was reviewed by the research team prior to data collection and further refined after the first two interviews to improve clarity and relevance. Each participant took part in a single interview, and no repeat interviews were conducted.

### 2.4. Data Analysis

All interviews were audio-recorded, transcribed verbatim, and analyzed in Hebrew. Quotations were later translated into English for publication following verification by the authors. The data were analyzed using Braun and Clarke’s [39] six-phase thematic analysis framework: (1) familiarization with the transcripts, (2) generating initial codes, (3) identifying potential themes, (4) reviewing and refining themes, (5) defining and naming themes, and (6) producing the final report. In practice, familiarization involved repeated reading of transcripts and initial note-taking; coding focused on identifying meaningful segments related to caregiving experiences; and theme development involved iterative comparison across participants, leading to the refinement and definition of themes. Coding was primarily data-driven, while also informed by the study’s theoretical framework, allowing both experiential and interpretive insights to emerge. The analysis was conducted iteratively and reflexively, with ongoing engagement with the data.

Two members of the research team were involved in the analytic process. Rather than coding independently to achieve agreement, researchers engaged in ongoing reflexive discussions to deepen interpretation, challenge assumptions, and refine the development of themes. These discussions were not intended to produce consensus, but to support a more nuanced and critically engaged analysis, in line with Braun and Clarke’s reflexive thematic analysis approach [39]. For clarity, participant quotations presented in Section 3 (Results) are labeled by parent identification number, corresponding to the demographic information in Table 1.

### 2.5. Reflexivity and Researcher Positioning

The first author, who conducted all interviews, is a healthcare professional with interdisciplinary training in nursing, health administration, and pharmacy, and prior clinical experience in pediatric intensive care and internal medicine. This background facilitated rapport building with participants and supported sensitive engagement with parents discussing emotionally complex experiences. At the same time, the researcher’s clinical perspective may have shaped the interpretation of parents’ narratives, particularly regarding healthcare systems, diagnostic processes, and family–provider interactions. To address this, reflexive awareness was maintained throughout the research process, with attention to how prior assumptions and professional experience might influence data interpretation.

The analytic process involved ongoing discussions among the research team, including co-authors with backgrounds in health research and experience in qualitative research. These discussions provided opportunities to guide the analytic process, challenge initial interpretations, consider alternative perspectives, and strengthen the analysis’s credibility.

## 3. Results

The thematic analysis identified five overarching themes: (1) child-related challenges, (2) the burden of parenting a child with FASD, (3) systemic barriers, (4) coping strategies and family strengths, and (5) unmet needs and parent recommendations. Table 2 summarizes these themes and their subthemes.

### 3.1. Child-Related Challenges

This theme captures the multifaceted neurodevelopmental, sensory, and behavioral characteristics of FASD as experienced by parents, emphasizing the daily unpredictability and functional gaps that shape family routines.

#### 3.1.1. Neurocognitive and Developmental Challenges

Parents described a broad spectrum of persistent neurocognitive and developmental difficulties beginning in early childhood and continuing into adolescence. These difficulties included global developmental delays, severe learning disabilities (especially in mathematics), memory impairments, and limited abstract reasoning. They also commonly reported impairments in executive functioning, including difficulty applying new knowledge, poor planning, and limited cognitive flexibility.

The mother of a 16-year-old boy stated: “Although he appears to be 16, internally, he is like a young child. There are things he will never be able to do… He has no sense of temporal continuity and no understanding of money” (P1). The father of another 16-year-old boy shared the following:

“He has an impaired perception of reality…There is also an impairment in connecting information and forming associations. This is one of the most dominant characteristics of this syndrome, the inability to connect and draw conclusions, which makes planning almost impossible”.

(P2)

#### 3.1.2. Sensory and Self-Regulation Issues

Parents frequently noted sensory overload, emotional dysregulation, and sleep disturbances. They described children who were easily overwhelmed and struggled to maintain emotional control during routine activities. One interviewee described, “As a baby, he cried excessively and was impossible to soothe. He had frequent and intense tantrums and would often wake up screaming and crying in the middle of the night” (P7). A father described, “His feelings are not well regulated. He may not say anything, even when he is in pain, or he may complain of pain even when it is something very minor” (P2). Another parent shared, “He cannot sit through an activity that requires patience or attention. Even a museum tour or a 2 h guided activity is impossible because he lacks the endurance and self-regulation needed” (P1).

#### 3.1.3. Behavioral Difficulties

Parents reported a wide range of behavioral challenges across settings, including aggression, tantrums, property destruction, compulsive shopping, resistance to authority, boundary testing, impatience, impulsivity, low frustration tolerance, inability to delay gratification, engagement in risky behaviors, lying, and running away from home, school, or residential care. Many children required constant supervision due to their limited danger awareness, vulnerability to exploitation, and smoking and alcohol consumption. Several participants described violent outbursts that placed family members at risk.

The mother of a 14-year-old recalled, “When he has a rage attack, he destroys everything: doors, furniture, everything. At home, there is no padded room like at school” (P8). Another mother recounted, “When he was 16, he took knives and wanted to kill us… He broke the glass door of his brother’s room and injured him” (P12).

### 3.2. The Burden of Parenting a Child with FASD

This theme reflects the substantial emotional, practical, and relational burden placed on caregivers. It highlights how the demands of FASD permeate parents’ daily lives, employment, health, and family dynamics.

#### 3.2.1. Parental Burden and Emotional Toll

The parents interviewed in this study described a profound emotional and parental burden associated with raising children with FASD. Many families faced complex psychological challenges, such as coping with adoption as well as FASD-related issues, including adopting children from diverse backgrounds, parental divorce, chronic illnesses, and difficulties in the parent–child relationship. The mother of an 18-year-old boy stated the following:

“My story is very complex from every possible angle, the different adoptions, the Ethiopian one, and the second. I became ill, I got divorced, … and in therapy. I have never found anyone who can understand all of these situations together”.

(P12)

Parents reported experiencing burnout, chronic stress, and heightened anxiety. They often attributed these experiences to the continuous demands of caregiving, the child’s complex cognitive and behavioral profile, and the inherent uncertainty of this unconventional parenting journey. One interviewee shared, “I cannot even take a shower without my phone next to me. You have to be ready all the time” (P2). The mother of a 16-year-old girl stated, “It felt like we could no longer live like this, watching her all the time, as if we were prison guards” (P4).

#### 3.2.2. Practical Costs of Parenting a Child with FASD

The parents reported significant impacts on their employment, finances, and personal well-being. They had to reduce their work hours, turn down promotions, or leave the workforce entirely. One interviewee said, “I had to quit my job for a year. He just cannot be left alone. Every moment requires supervision and intervention; otherwise, something will go wrong” (P8). Another interviewee reported the following:

“It is a full-time job just being her mother. I drove her to therapy three times a week, to social skills groups, to volunteer activities, and to every appointment. My whole life revolved around her schedule, and there was no time left for myself”.

(P11)

One interviewee described the following:

“I became self-employed and started working from home because I could be needed at any moment. It affected my relationship. It was impossible; we decided to separate because we could not… we could not hold the family together”.

(P2)

Some parents noted declining physical and mental health and restrictions on travel or professional opportunities. One interviewee noted, “We considered going abroad for an assignment and to work there, but he needs special education in Israel” (P1). Another mother explained, “I need to go abroad for surgery and will require a few weeks of recovery, but because of his special needs, I have no one to leave him with in Israel” (P5). Reflecting on the personal toll, another parent shared, “I think I have become much more anxious and under psychological stress. I have had periods of high blood pressure, and I do believe it affects my health” (P8).

### 3.3. Systemic Barriers

This theme describes parents’ encounters with educational, medical, and welfare systems that lack adequate knowledge, resources, and awareness of FASD, resulting in fragmented services, misinterpretation of behaviors, and delayed access to support.

#### 3.3.1. Challenges with the Education System

Parents reported that schools often lacked an understanding of FASD, held unrealistic expectations, and were unwilling to learn about the syndrome. One interviewee said that “the school blamed him… They punished him for things he could not control [because] the school just did not get it” (P3). Another mother said, “It felt like they were always trying to kick him out. Even in special education, there was no patience, no understanding” (P10).

The parents emphasized the lack of educational frameworks that integrate emotional, behavioral, and cognitive needs. One interviewee explained, “The problem with special education is that it typically addresses either emotional–psychological issues or learning disabilities, without providing a comprehensive framework that supports behavioral, emotional, and cognitive difficulties simultaneously” (P9). Another mother noted, “Ultimately, within the Ministry of Education, there is no framework tailored for children with FASD” (P3).

#### 3.3.2. Limited Awareness Among Professionals

The parents consistently encountered limited professional knowledge across the health care, welfare, and education sectors. They reported that many professionals did not recognize the condition. They misinterpreted the child’s difficulties or offered generic parenting guidance rather than interventions appropriate for FASD. One interviewee described, “The clinic director, who is a physician, ended up learning everything she needed to know through our case” (P4). Another mother elaborated, “It is not only physicians who do not know what FASD is… Even our senior psychiatrists did not know” (P8). One interviewee said, “All the therapists we went to did not know, did not understand what FASD is, and did not know how to speak this ‘language’” (P2).

#### 3.3.3. Diagnostic Process Barriers

Parents described a long, fragmented, and often frustrating diagnostic process, influenced by clinicians’ unfamiliarity with FASD and reluctance to diagnose, a lack of multidisciplinary teams, bureaucratic obstacles, and inconsistent diagnostic protocols. One interviewee explained, “Only one doctor we met really knew what FASD was. The rest either dismissed it or admitted they did not know how to diagnose it” (P4). Another mother shared, “I know of parents who are still going through a very long process and are not succeeding because, in this country, there are almost no doctors who can diagnose this” (P7).

#### 3.3.4. Lack of Specialized Services

Parents emphasized the lack of FASD-specific services and the need to navigate care independently. Many families reported relying on services they found through word of mouth, which they considered effective in addressing their children’s unique needs. The mother of a 15-year-old boy explained: “I am not talking about the money. Just give me a professional who can help me, first of all. But there is no one, no one to help” (P10). Another mother revealed, “I take my son to another city. I found [the services] through a parents’ association, which they wholeheartedly recommend” (P7).

Even when families identified helpful services, access was limited due to geographic, financial, and systemic barriers. One interviewee recounted, “His treatments are essential and long-term, but he does not meet several of the eligibility criteria for reimbursement, leaving me to cover all the costs myself” (P2).

### 3.4. Coping Strategies and Family Strengths

This fourth theme highlights how families adapt to ongoing challenges, drawing on personal resourcefulness, informal support networks, and recognizing their children’s strengths.

#### 3.4.1. Support Systems

Parents frequently emphasized the importance of family, friends, and community support in coping with the daily challenges of raising a child with FASD. Interviewees reported that this informal support was their primary source of help, both practically and emotionally. One interviewee explained: “My extended family supports me both practically and emotionally; there is empathy” (P8). Another mother shared, “It is a whole community helping to raise her. For example, people report to me if they see her, because they understand it is important” (P4).

Parents also highlighted the insufficient availability of formal psychological and respite services. One interviewee said, “We received mental health support from a 20-year-old social worker who is not a parent and does not know about FASD, so it is not helpful.” She added, “We do not have mental health support. Mostly, I am the one supporting others” (P4). Another mother said the following:

“Yes, I would be glad if there were more support frameworks. What really helps me emotionally is mostly other parents of kids like mine that I know from the past. We once had a support group that met for 10 sessions, and it was beneficial to sit and talk. I wish there were more opportunities like that”.

(P6)

#### 3.4.2. Children’s Strengths

Despite these challenges, the parents identified their children’s notable strengths, including creativity, social warmth, curiosity, and athletic abilities. One interviewee described her son: “He is a child with much energy, very curious, loves to see, climb, explore, and collect things” (P1). Another mother said, “He is a real charmer; he captivates people instantly and connects easily with both adults and peers” (P6). Another mother stated, “She is very creative, loves art, and has been in many art therapy sessions” (P3). A fourth mother shared, “He is very athletic and excels in almost all sports” (P10).

### 3.5. Unmet Needs and Recommendations

The final theme highlights gaps in diagnostic services, educational supports, and long-term care, as well as parents’ concrete recommendations for improving systems and services for children with FASD.

#### 3.5.1. Unmet Needs

The interviewed parents consistently emphasized the urgent need for formal recognition of their child’s FASD-related needs across all official systems. In practice, even with a confirmed FASD diagnosis, families rarely received entitlements linked explicitly to the condition. One interviewee explained: “There is financial support from the National Insurance, but it is not for FASD; it is for supervision needs” (P5). Another mother noted, “There is no recognition of FASD within the welfare system, unlike the recognition granted to individuals with autism” (P1).

Parents also expressed concern about the absence of adult services, including housing, financial management, supported employment, and lifelong supervision. Some parents described their efforts to establish legal and financial safeguards for the future, while others noted the lack of inheritance mechanisms suited to the unique needs of these individuals.

One interviewee explained that his son “will not be completely independent… He will always need some kind of supervision, supervision that does not exist now.” He added, “If I leave him an inheritance, he could spend it in a minute,” and expressed deep concern about his child’s future: “I have no idea how he will survive… I cannot place him in a hostel for people with autism, it does not fit him; but, at the same time, he cannot live on his own” (P2). One interviewee shared that her son with FASD asked her “to go to National Insurance now and tell them to give his allowance to his brother so he can pay my rent, and I will not end up on the street” (P11).

#### 3.5.2. Parents’ Recommendations

All parents emphasized the need to establish a dedicated medical center for the diagnosis and treatment of individuals with FASD, noting that such centers already exist abroad. They also emphasized the importance of raising awareness among professionals and educators, developing a comprehensive support framework for families, advocating for formal recognition of the syndrome, and promoting legislation to establish permanent disability status and support mechanisms that extend beyond adolescence. One interviewee expressed her hopes that “when the FASD association navigates the way, [it will] create support pathways for families and develop solutions and mechanisms to support children with the syndrome beyond the age of 21” (P4).

## 4. Discussion

This study explored the lived experiences of adoptive parents raising children with FASD in Israel. While core caregiving challenges, such as chronic vigilance and emotional exhaustion, are well-documented internationally [25,32], the findings demonstrate how these experiences are intensified and reshaped within the Israeli context. Parents navigated a service environment characterized by limited professional awareness, fragmented diagnostic pathways, and a lack of formal recognition across healthcare, education, and welfare systems. As a result, caregiving extended beyond daily management to include sustained efforts to obtain a diagnosis, access services, and coordinate support. In this context, parents assumed expanded roles as advocates, coordinators, and knowledge brokers within fragmented systems.

**Figure 1:**
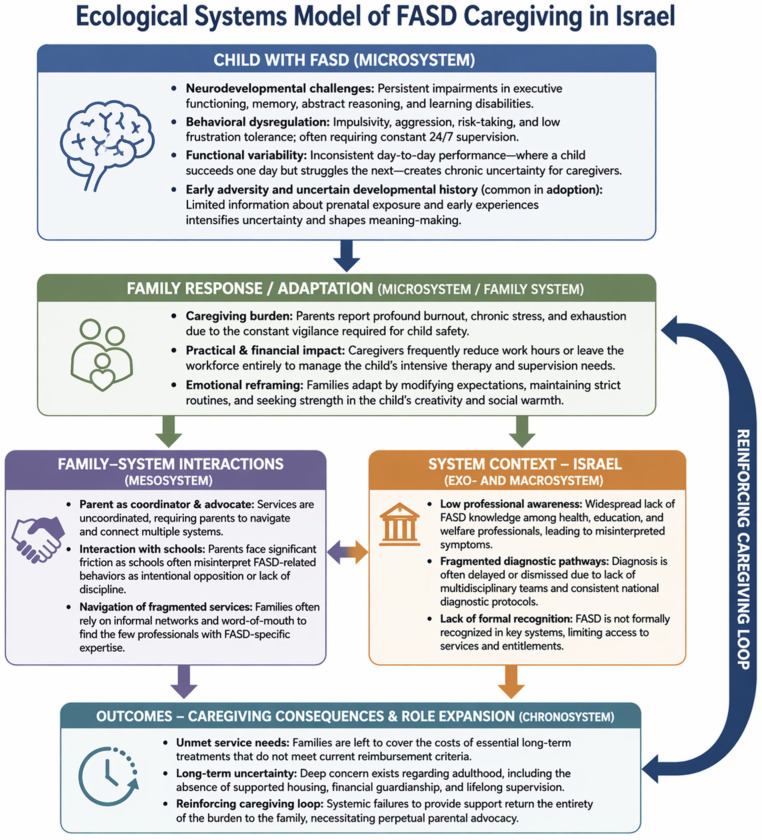
Ecological systems model of FASD caregiving in Israel. The model synthesizes the study findings and illustrates how child-related neurodevelopmental challenges, family adaptation processes, and system-level factors interact across ecological levels to shape caregiving experiences. The reinforcing loop highlights how systemic limitations and family responses mutually sustain and intensify caregiving demands over time.

As illustrated in Figure 1, caregiving experiences are shaped through dynamic interactions across ecological levels, including processes at the micro level (family interactions), meso level (interactions between families and service systems), and macro level (broader policy and societal contexts). These interacting levels generate a reinforcing cycle of burden and adaptation. From this perspective, caregiving burden in FASD cannot be understood solely in terms of child characteristics, but is co-produced through interactions among the child’s neurodevelopmental profile, family adaptation processes, and broader systemic conditions. In low-recognition environments, systemic limitations do not merely influence family life but actively reorganize it. Accordingly, family adaptation is shaped not only by the child’s needs but also by the degree of diagnostic legitimacy and institutional coordination available in the surrounding systems.

This perspective further points to underlying processes that extend beyond the descriptive themes. In particular, the patterns observed reflect system-level failure and diagnostic invisibility. These processes are not independent but mutually reinforcing: systemic barriers intensify caregiving burden and require parents to assume expanded roles, thereby sustaining the cycle of demands identified across themes. In turn, fragmented services and limited recognition of FASD contribute to ongoing uncertainty and place additional interpretive and coordination demands on parents.

The adoptive context further shapes, and in some cases intensifies, these dynamics. Parents often enter caregiving with limited or uncertain information regarding prenatal exposure and early developmental history, complicating both diagnosis and understanding of the child’s needs [40,41]. Combined with early adversity, often including histories of neglect, institutional care, or exposure to unstable environments, this requires navigation between trauma-related, attachment-based, and neurodevelopmental explanations [42]. These overlapping factors complicate both diagnosis and caregiving, as parents must interpret behaviors that may stem from multiple, interacting sources. Consequently, caregiving involves ongoing interpretation and meaning-making, highlighting adoptive parenting in FASD as a process shaped by uncertainty and distinguishing it from other caregiving contexts [43]. In contrast to studies of biological parents, which often address issues related to prenatal disclosure and parental responsibility [44], the present findings suggest that adoptive parents face additional challenges related to limited access to early developmental and prenatal histories. This lack of information may further complicate the diagnostic process and contribute to ongoing uncertainty in understanding the child’s needs.

### 4.1. Neurodevelopmental and Behavioral Challenges

The parents in this study consistently described the neurodevelopmental and behavioral difficulties characteristic of FASD, including deficits in executive functioning, attention, learning, and emotional regulation. These observations align with established evidence that FASD presents with heterogeneous and persistent neurocognitive impairments [32,45]. The parents also emphasized the day-to-day variability in functioning: a child may perform a task successfully one day but struggle with it the next. Such inconsistencies have also been reported in studies from Canada and Australia [8,46]. These fluctuations contribute to chronic uncertainty, as described in previous studies, and challenge parents’ expectations across developmental stages [47].

Behavioral difficulties, including impulsivity, aggression, and risk-taking, are a prominent concern and often require constant monitoring. These findings align with the international literature, which demonstrated the high levels of externalizing behavior among children with FASD and the considerable safety risks that accompany such behaviors [24,30]. The parents in our study described adolescence as an especially challenging period, consistent with developmental patterns reported in previous research [18]. Some parents also referred to additional health-related concerns; however, these were not detailed and did not emerge as a central theme in the interviews.

### 4.2. Emotional and Practical Demands of Caregiving

Raising a child with FASD had substantial emotional, physical, and practical consequences for parents. Participants described persistent stress, exhaustion, and a sense of constant vigilance, consistent with research documenting the caregiver burden of FASD [25,48]. Many participants noted the strain on family relationships, limited personal time, and reduced capacity to meet the needs of other family members.

The parents also expressed ongoing worry about their child’s future, including concerns about independence, safety, and long-term support. These findings align with previous studies that found caregivers reported chronic uncertainty and fear regarding their child’s developmental trajectory [16,30]. In our study, emotional strain was exacerbated by limited access to professional guidance, leaving many parents feeling that the responsibility for understanding and managing the condition rested heavily on them.

### 4.3. Systemic Barriers Across Education, Health Care, and Diagnostic Services

A major theme that emerged from our study involved parents’ encounters with systems unequipped to support children with FASD and their families. Consistent with international findings, participants reported limited professional knowledge of FASD, diagnostic delays, and inconsistent assessments across healthcare settings [28,49]. As explored in other studies, many of our participants experienced long and fragmented diagnostic pathways, reflecting global concerns about underdiagnosis and the lack of multidisciplinary evaluation teams [50,51].

The parents in our study also reported substantial difficulties with the education system, where FASD-related behaviors were often misinterpreted as intentional or oppositional. Studies from the United Kingdom, Australia, and Canada have documented similar patterns, in which a lack of teacher training contributed to inappropriate disciplinary responses and insufficient accommodations [46,52,53]. For the families in our study, the inconsistent implementation of individualized education plans further contributed to frustration and school-based stress. Collectively, these systemic barriers increased the parents’ emotional load and required them to invest significant time and effort advocating for appropriate services. Similar challenges have also been described in resource-constrained settings, where caregivers navigate complex child needs within fragmented or under-resourced systems [33].

### 4.4. Coping Efforts and Family Strengths

Despite these significant challenges, the parents reported a range of coping strategies that helped them manage their daily lives. These include maintaining structured routines, modifying expectations, seeking support from peers or family, and identifying their child’s strengths. Such strategies mirror international findings that caregivers of children with FASD develop considerable expertise and adaptive skills over time [20,32,48]. However, the findings also highlight an important nuance. While these strategies may be effective in the short term, they appear insufficient to address the multiple and overlapping demands identified in this study. In contrast to caregivers in contexts where diagnostic and support pathways are more established, participants in this study often navigated the combined challenges of adoption-related uncertainty and fragmented service systems. This intersection required parents to engage not only in caregiving but also in ongoing advocacy and coordination efforts. Consequently, many participants described limitations in the effectiveness of their coping strategies in the absence of adequate professional support. This aligns with broader research indicating that family resilience alone cannot compensate for systemic gaps and that sustained external support is essential to prevent caregiver burnout [45]. The present findings extend this understanding by illustrating that individual and family resources shape how coping occurs, not only by the level of coordination and support within surrounding service systems.

### 4.5. Unmet Needs and Implications for Support

The parents identified clear and consistent unmet needs across diagnostic, educational, and therapeutic domains. Their recommendations include establishing multidisciplinary diagnostic centers, enhancing professional training, improving coordination between systems, and expanding parent support and respite services.

These parent-identified priorities align with international guidelines for improving FASD services [22,54] and point to a set of concrete, actionable policy tasks that could guide the development of FASD-informed services in Israel and similar contexts. First, there is a need for national, evidence-based diagnostic guidelines for FASD, adapted from established international models. These include widely used national diagnostic guidelines, such as those from Canada [55], Australia [56], and the United States [57]. These guidelines should support multidisciplinary assessment and promote consistent diagnostic practices across health maintenance organizations. As an initial step, regional pilot diagnostic centers with centralized expertise and clear referral pathways could be established.

Second, diagnostic improvements must be accompanied by targeted professional training. Limited knowledge among health, education, and welfare professionals contributes to delayed diagnosis and increased parental burden. National policy should therefore integrate FASD-specific training into pre-service education and continuing professional development, with an emphasis on the neurodevelopmental nature of FASD, functional understanding of behavior, and developmentally appropriate expectations.

Third, international models emphasize the value of integrated, lifespan-oriented services. In countries such as Canada and the United States, specialized FASD centers combine diagnosis with individualized intervention planning, parental guidance, and ongoing care coordination. Adapting this approach locally would involve assigning a single care coordinator to align educational, therapeutic, and welfare services.

Fourth, the findings highlight a marked lack of adult services for individuals with FASD. Policy planning should extend beyond childhood to include supported housing, supervised employment, financial guardianship, and long-term supervision. Preparation for adulthood should begin in adolescence through a structured transition process involving families and service systems.

Finally, parents’ accounts point to the need for formal recognition of FASD within disability and welfare legislation, similarly to existing frameworks for autism and intellectual disability. Such recognition would ensure consistent access to financial support, respite care, and caregiver assistance, reducing families’ reliance on informal solutions and the need for sustained advocacy.

### 4.6. Strengths, Limitations, and Future Studies

A key strength of this study is its focus on adoptive parents raising children with FASD, a group of particular relevance given the substantially higher rates of prenatal alcohol exposure among children who enter adoption and out-of-home care. In addition, the use of in-depth interviews enabled parents to provide rich, detailed accounts of their daily challenges and navigation of service systems, offering insights that are often not captured in quantitative research.

Nevertheless, several limitations should be considered when interpreting the findings. Although the sample size is appropriate for a qualitative inquiry, participants were recruited primarily through parent support groups and snowball sampling. This recruitment strategy may have introduced sampling bias, as participants were likely to be more engaged, resourceful, and connected to support networks than other adoptive parents of children with FASD. As a result, the findings may overrepresent active coping strategies, help-seeking behaviors, and system navigation, while potentially underrepresenting the experiences of more isolated or less supported families. As with qualitative research, the findings are not intended to be statistically generalizable but rather to provide in-depth insights into the experiences of a specific group of participants.

Another limitation relates to variability in diagnostic pathways in Israel. Due to the limited availability of standardized, multidisciplinary diagnostic services, parents’ reports of their child’s diagnosis may reflect differing levels of clinical certainty or assessment quality. While this does not detract from the validity of their lived experiences, it introduces heterogeneity in the diagnostic context that should be taken into account when interpreting the findings.

Future research should build on these findings by examining diagnostic and educational practices in Israel to better understand the systemic barriers that parents encounter. Studies involving healthcare and education professionals are needed to clarify knowledge gaps and inform training. Longitudinal research could further illuminate how challenges and coping strategies evolve over time, particularly in adoptive families facing complex and dynamic caregiving demands.

## 5. Conclusions and Clinical Implications

This study illuminates the experiences of adoptive parents raising children with FASD and highlights the persistent neurobehavioral, emotional, and functional challenges that shape family life. Parents describe significant daily demands, inconsistent understanding from professionals, and concerns about their children’s future independence. At the same time, they emphasize their children’s strengths and the importance of tailored support. These findings underscore the need for clinicians, at the micro level, to actively incorporate parents’ perspectives into assessment and intervention and to recognize the chronic, fluctuating nature of FASD-related difficulties. The results also highlight the importance of strengthening professional training in FASD across healthcare, education, and welfare systems, at the meso level, to support more accurate recognition of neurodevelopmental impairments and more appropriate responses to children’s behavior. In addition, developing coordinated multidisciplinary service pathways, including the potential role of care coordinators, may help reduce the burden on families required to navigate fragmented systems independently and support both immediate challenges and long-term planning for children and adolescents with FASD across developmental stages (chrono level).

This study has important clinical and policy implications, as it highlights the experiences of adoptive parents raising children with FASD, a high-risk group that remains underrepresented in research and underserved in practice. The findings demonstrate how fluctuating neurodevelopmental and behavioral difficulties, combined with limited professional awareness and fragmented diagnostic pathways, shape daily life and increase parental burden. These findings further underscore the need for improved diagnostic infrastructure, FASD-informed educational practices, and coordinated multidisciplinary services at the macro level. Furthermore, the study makes a conceptual contribution by demonstrating how ecological and family systems perspectives help explain the interactions among child characteristics, family adaptations, and systemic barriers, thereby advancing understanding of FASD in adoptive contexts.

## Figures and Tables

**Table 1:** Participants’ Sociodemographic Characteristics.

Parent ID	Parent Gender	Parent Age (Years)	Marital Status	Parent Education	Number of Children (Total/ Adopted)	Child’s Gender	Child’s Age (Years)	Age at Adoption	Child’s Age at Diagnosis (Years)	Country of Adoption
P1	Female	54	Married	MA	3/1	Male	16.0	2.5 years	4.0	Ukraine
P2	Male	51	Divorced	MA	1/1	Male	16.0	2.5 years	4.0	Russia
P3	Female	51	Married	MA	2/1	Non-binary	10.5	1 year 8 months	5.0	Russia
P4	Female	50	Married	MA	3/1	Female	16.0	1.5 years	10.0	Ukraine
P5	Female	60	Divorced	High school/professional course	1/1	Male	12.0	1 year 5 months	6.0	Russia
P6	Female	54	Single	BA	1/1	Male	13.0	3 years	3.5	Russia
P7	Female	62	Married	BA	4/1	Male	9.0	3 days	5.5	Israel
P8	Female	56	Single	MA	2/1	Male	14.5	2 years	9.0	Russia
P9	Male	Missing	Married	MA	3/3	Male	15.0	2 years	4.5	Ukraine
P10	Female	53	Married	MA	3/3	Male	15.0	2 years	4.5	Ukraine
P11	Female	58	Divorced	MA	2/2	Female	15.0	1.5 years	8.5	Russia
P12	Female	53	Divorced	Teaching certificate	2/2	Male	18.0	3 months	16.0	Israel

Note. “Total/Adopted” indicates the total number of children in the family, followed by the number of adopted children. In families with more than one child with FASD, the table refers to one focal child per family. Participants P9 and P10 are parents of the same child and were interviewed together.

**Table 2:** Summary of Themes and Subthemes.

Theme	Subtheme
1. Child-related challenges	a. Neurocognitive and developmental challenges b. Sensory and self-regulation issues c. Behavioral difficulties
2. The burden of parenting a child with FASD	a. Parental burden and emotional toll b. Practical burden of parenting a child with FASD
3. Systemic barriers	a. Challenges with the education system b. Limited awareness among professionals c. Diagnostic process barriers d. Lack of specialized services
4. Coping strategies and family strengths	a. Support systems b. Children’s strengths
5. Unmet needs and parent recommendations	a. Unmet needs b. Recommendations for improving support and services

## Data Availability

The data presented in this study are not publicly available due to ethical and privacy restrictions, as they contain sensitive information from qualitative interviews with participants. Data may be available from the corresponding author upon reasonable request and subject to ethical approval.
